# Computing the human interactome

**DOI:** 10.1063/4.0000966

**Published:** 2025-10-27

**Authors:** Jing Zhang, Ian Humphreys, Jimin Pei, Qian Cong

**Affiliations:** 1 University of Texas Southwestern Medical Center, Dallas, TX, USA; 2 University of Washington, Seattle, WA, USA

## Abstract

Protein-protein interactions (PPI) are essential for biological function. Recent advances in coevolutionary analysis and Deep Learning (DL) based protein structure prediction have enabled comprehensive PPI identification in bacterial and yeast proteomes, but these approaches have limited success to date for the more complex human proteome. Here, we overcome this challenge by 1) enhancing the coevolutionary signals with 7-fold deeper multiple sequence alignments harvested from 30 petabytes of unassembled genomic data, and 2) developing a new DL network trained on augmented datasets of domain-domain interactions from 200 million predicted protein structures. These advancements allow us to systematically screen through 200 million human protein pairs and predict 18,316 PPIs with an expected precision of 90%, among which 5,578 are novel predictions. 3D models of these predicted PPIs nearly triple the number of human PPIs with accurate structural information, providing numerous insights into protein function and mechanisms of human diseases.

